# Management of severe low back pain with a focused vibro‐percussion wave treatment: A case report

**DOI:** 10.1002/ccr3.6054

**Published:** 2022-07-22

**Authors:** Norah M. Alsalamah, Lee Bartel

**Affiliations:** ^1^ KKT Orthopedic Spine Center Qassim Saudi Arabia; ^2^ Neuro Spinal Innovations Research Department Mississauga ON Canada; ^3^ University of Toronto Toronto ON Canada

**Keywords:** case report, Khan Kinetic Treatment, KKT, low back pain, low frequency sound, sound wave therapy, spinal stenosis, vibration therapy, vibroacoustic

## Abstract

A 49‐year‐old male with severe low back pain (LBP) showed multilevel disc bulges with spinal stenosis. After 18 novel low‐frequency sound wave treatments, initial VAS pain score of 9.5 reduced to 2.5 and the Rolland‐Morris score of 13 reduced to 3. MRI showed some resolution of L3–L4 and L4–L5.

## Graphical abstract

Focused vibro‐percussive sound wave stimulation to the spine known as Khan Kinetic Treatment (KKT) has in this case, and previously through RCT studies, demonstrated improvement in pain and function with no adverse events. KKT is currently available in 10 countries.

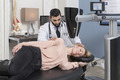

## 1 INTRODUCTION

Back pain (BP) is a leading cause of years lived with disability,^1^ with an estimated 70–85 percent of the population to experience BP at some point in their lives.^2^ In North America, disability from BP increased by 14 times the rate of population growth between the years 1950 and 1980.^2^ When measuring overall disease burden, these trends have also occurred on a global scale in all continents, but significantly so in areas such as South Asia, East Asia, North Africa, and the Middle East. Poor education about workplace ergonomic and a lack of effective medical management is thought to be among the main causes of the increase in overall burden.^1^

The biggest challenge in treating BP is determining the actual development of the injury. Spine stability is based on three components: (1) vertebrae and facet joint, ligament, and intervertebral disks; (2) muscles and tendons surrounding the spine; (3) neural systems that coordinate the active subsystem.^3^ A disruption in any one of these three components leads to instability of the spine and injury and pain.^3^ Currently most evidence‐based treatment modalities focus on a combination of pain medications, exercise, and psychological interventions. Although a minority of patients with chronic BP may qualify for surgery, serious side effects and high costs are an impediment.

A factor in low back pain (LBP) may be obesity resulting in stress on the musculoskeletal system.^4^ Associated with obesity may also be an infiltration of fat into the multifidus and erector spinae muscles and vertebral modic changes.^5^ Because of the association of obesity and severe LBP, bariatric surgery is conducted in some cases with positive results.^6^ However, there is also a known effect on some patients that a gastrectomy is followed by increasing LBP resulting from the change in intra‐abdominal pressure, spinal loading, and a decrease in stability of the spine.^7^

Consequently, alternative treatment modes are being explored. One novel treatment being used successfully for many patients is the KKT Treatment (Khan Kinetic Treatment now also known as SONiK Treatment). It is a unique treatment, but not experimental. To date it has been used in some 1.5 million treatments in 23 clinics in 10 countries and there is only one clinic in North America and one in Europe so it is still relatively little known. The treatment is novel in that it does not merely use a single percussive thrust like Atlas Orthoganal, does not use a repetitive nonfrequency‐specific sound blast like Shockwave, but uses a precisely dosed (amplitude and frequency), multiple frequency, directionally precise (X‐ray analysis determined), and at spine‐related diagnostically determined application locations. This noninvasive approach provides orthopedic spinal treatment with focused sound waves. The KKT device delivers accelerated low‐frequency kinetically directed impulses (ALKINDI) meaning: “accelerated”—a rapidly increasing frequency curve, “low frequency”—at the lowest level of human audibility in the 10–100 Hz range, “kinetically directed”—very specifically angled and directed at vertebrae targets, and “impulses”—sound‐initiated percussion waves, focused through a stylus onto particular locations on the spine. This sound wave treatment is a safe application that has demonstrated the ability to decrease pain, reduce anxiety, reduce the symptoms of illness, and generally promote health in many patients.^8^ Research has shown that the musculoskeletal effect of this novel sound wave treatment not only results in pain reduction and spinal alignment but also includes an increased mRNA expression of key proteins for spinal health and a thus providing a cellular environment conducive to ligament repair.^8^, ^9^, ^10^, ^11^

Low back pain is a major cause of patients' suffering and increased health care costs. Treatment for LBP should ideally control pain perception, improve alignment of the spine, and stimulate the healing of ligamentous structures. Research into this sound wave treatment has theorized that the KKT treatment addresses these crucial mechanisms for effective back treatment at the very core by: (1) stimulating intervertebral disc biosynthesis; (2) Correcting abnormal intervertebral joint movement (mean axis of rotation, MAR); (3) Gating pain transmission by activating key circuitry in the spinal cord; (4) Minimizing asymmetrical loads on the spine by normalizing the paraspinal muscles; and (5) increasing coordination of muscle groups that play a critical role in stabilization of the spine.^8^, ^9^ The goal of the above is ultimately to improve the management approach for pain to help reduce the unnecessary suffering and costs of such pain. The following case report of an adult patient who presented with LBP illustrates the effect of the KKT treatment.

## 2 CASE PRESENTATION

The patient was a 49‐year‐old man who presented to the KKT Clinic at Qassim with severe LBP radiating to both lower limbs. He walked into the clinic with an antalgic gait, an abnormal pattern of walking secondary to pain that ultimately causes a limp, and was holding walls while changing direction. His history showed that he developed BP in 2016 and his BP became more severe in 2018 when he weighed 130 Kg. It affected his work to the extent that he was off work for a year. In an attempt to relieve the BP, he underwent a gastrectomy in February 2020. Before the surgery, his VAS score was 6 or 7/10 and after the surgery it became 9/10. He lost 35 kg but the increase in BP following the surgery caused him to stay in bed for a month. Post surgery, he also developed a deficit in vitamin B12, folic acid, and iron. He took supplements and his vitamin and mineral values returned to normal. When he presented at the KKT clinic, he had complaints of LBP radiating to both lower limbs, pain aggravated by standing, sitting, and walking. The patient reported no urinary or defecation incontinency. He experienced psychological stress, a sleep disorder, and frequent headaches, but had no history of other health disorders or injuries. Prior to presenting at the clinic, he tried to manage his symptoms using pain medications and standard treatments including physical therapy, osteopathy, and massage but none of these improved his pain or sleep. The patient has given informed written consent for his images and other clinical information to be reported in this journal.

## 3 PHYSICAL EXAMINATION AND DIAGNOSIS

**FIGURE 1:**
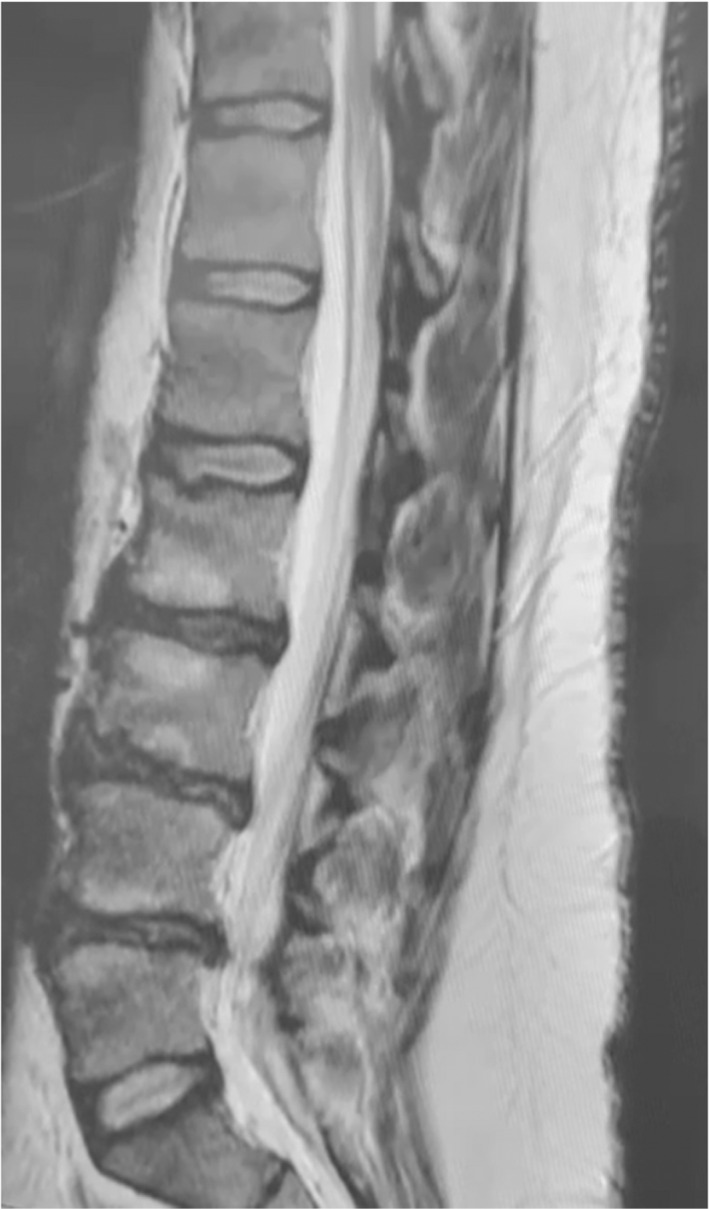
MRI of the lumbar spine before treatment showing bulging of the discs between L5 and L2 region

**FIGURE 2:**
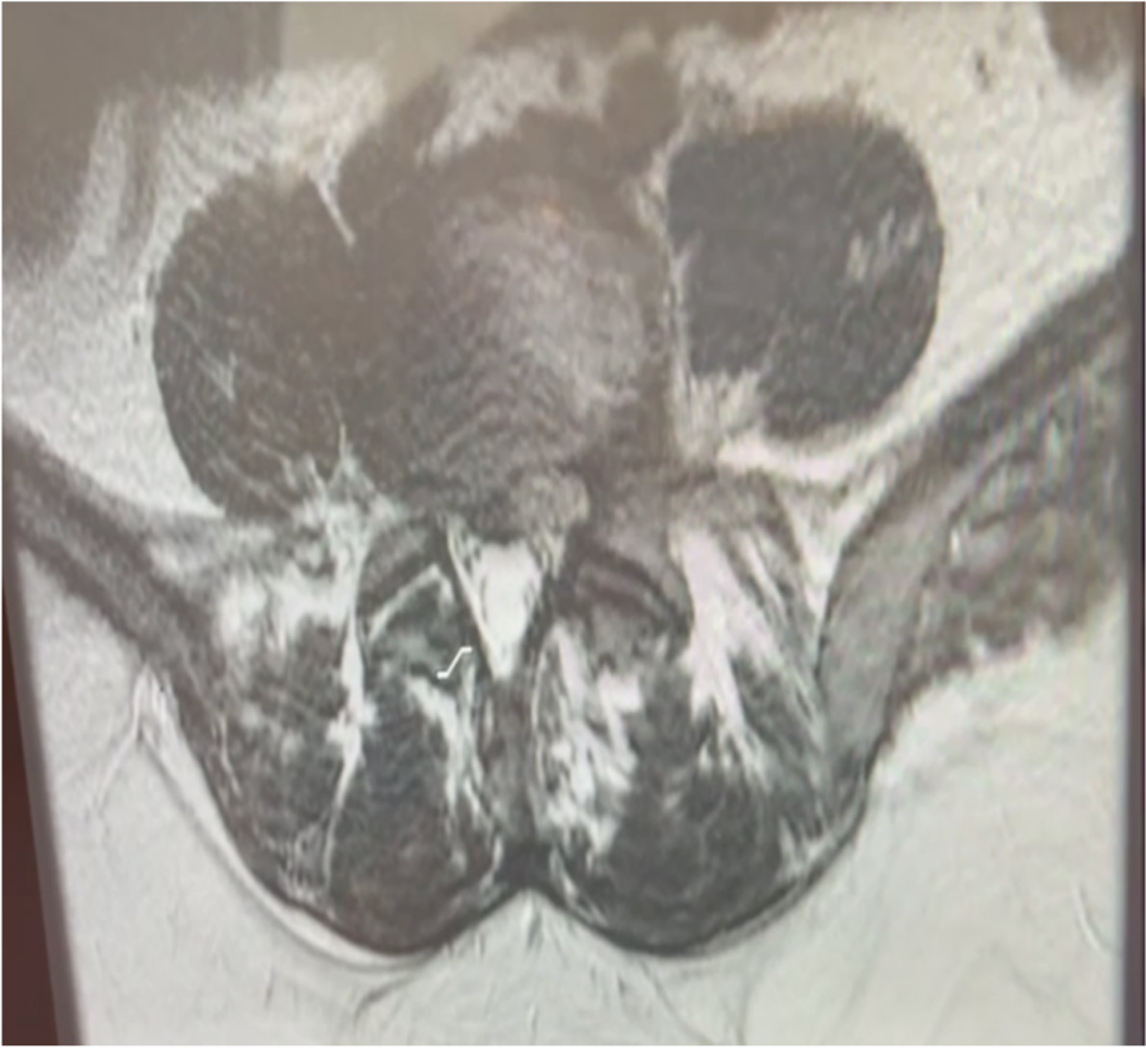
Axial MRI view of L5‐L4 before treatment

At the initial examination, the patient had a Visual Analogue Scale (VAS) for pain score of 9/10 and Roland‐Morris (RM) cumulative score of 13 points. Magnetic resonance imaging (MRI) with a superconductive 1.5 Tesla magnet of the lumbar spine showed the presence of multilevel disc bulges with mild spinal canal stenosis (Figures 1 and 2). Specifically, at L2/3 through L4/5 a diffuse posterior disc bulge is seen effacing the epidural fat indenting the thecal sac and encroaching on lateral neural recesses and foramina. A schmorl node is evident in L2. Some lumbar straightening is observed denoting a muscle spasm. There is a normal alignment of the lumbar spine with no signs of instability detected and normal bony texture of the lumbar vertebrae with no focal lesions identified. Spondylodegenerative changes of the lumbar spine that are evident in the MRI include: (1) reduced disc heights, (2) discs desiccation, (3) marginal osteophytosis, (4) facet osteoarthropathy, (5) sub‐end plate degenerative marrow changes,

Other observations include normal site, shape and intensity of the conus medullaris and cauda equina roots, no significant bony canal tightness, and normal pre and paravertebral soft tissues.

The clinicians in this case considered other diagnoses including a multilevel lumbar disc disorder with radiculopathy stemming from the lumbosacral region.

**FIGURE 3:**
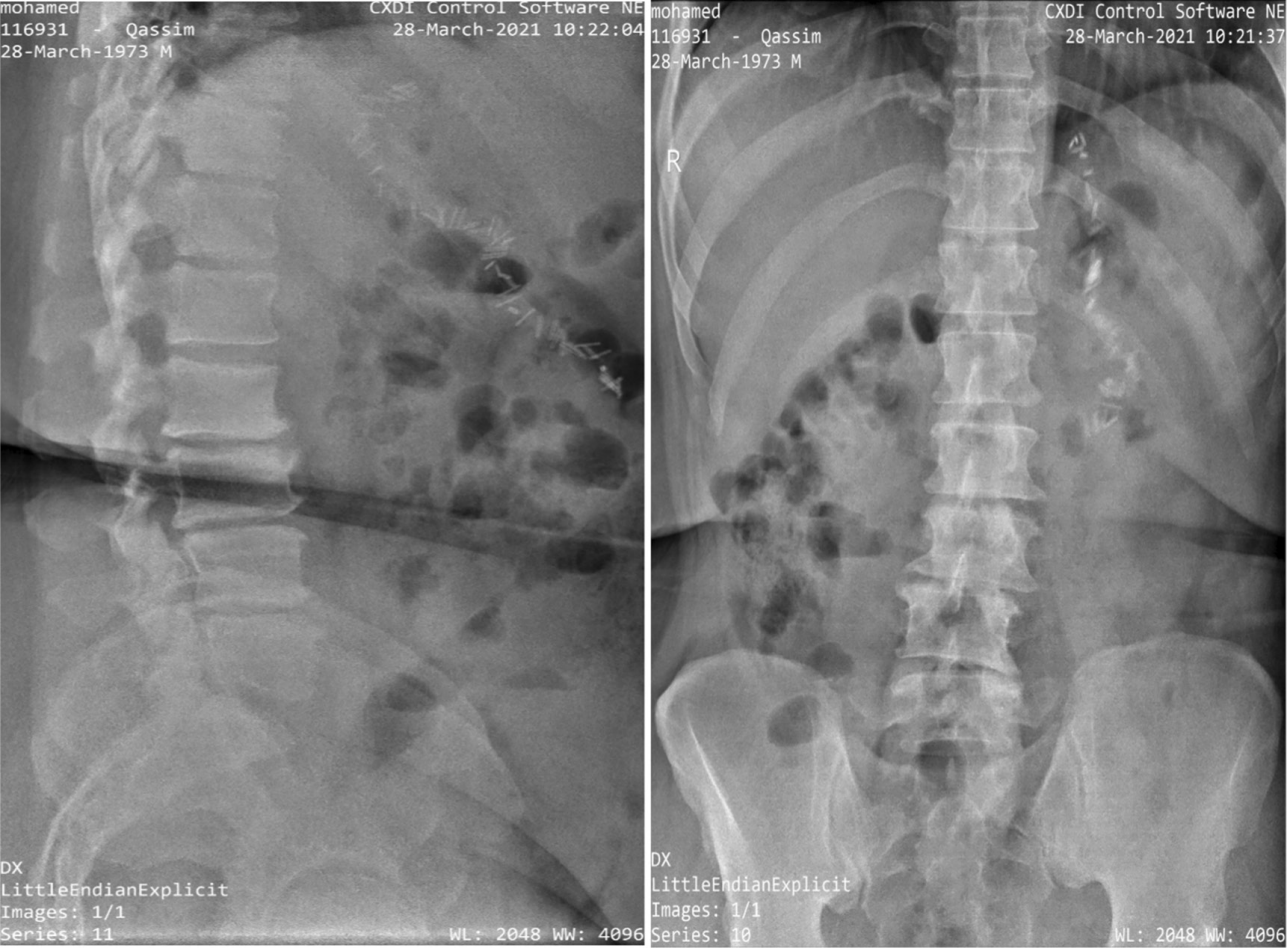
Thoracic X‐ray before treatment

A physical examination was performed to determine the severity of symptoms and possible causes. Palpation of the patient revealed severe tenderness in the mid‐thoracic and lumbosacral spine area, moderate to severe limitation of trunk flexion due to pain, and positive straight leg raise (SLR) test on both sides. Because of the mid‐thoracic pain, X‐rays were taken of the thoracic area but thoracic MRI was not required because clinicians determined that the thoracic X‐rays did not reveal a potential problem (Figure 3).

As part of the diagnosis and before each treatment a series of physical and neurological tests were conducted: (1) range of motion (ROM) of the cervical spine; (2) degree of tilt (to a quarter degree with a set of calipers) of the shoulder and pelvis in the coronal plane; (3) discrepancy of supine leg length; (4) coordinated response to resistance of arm and leg; and (5) tender lesions associated with deep spinal palpation. If results of the assessment before the treatment showed a departure from normal, the indicated assessments were repeated after each treatment application to determine response. At the initial examination the patient presented with: (1) normal cervical range of motion (ROM) to both sides; (2) the shoulder tilt deviation to the right by one degree and the pelvic tilt deviated to the right by 0.5 degrees; (3) arm coordination response to resistance was (5/5) bilaterally; (4) leg coordination was reduced on the left (3/5) and the right (4/5); (5) supine leg length was 1 cm shorter on the right side compared to the left side; and (6) and painful tender lesions were found at 6 points along the spine. The potential for surgery was discussed with the patient but he was determined to pursue this noninvasive novel treatment before making the decision for surgery.

## 4 TREATMENT PLAN AND FOLLOW‐UP

**FIGURE 4:**
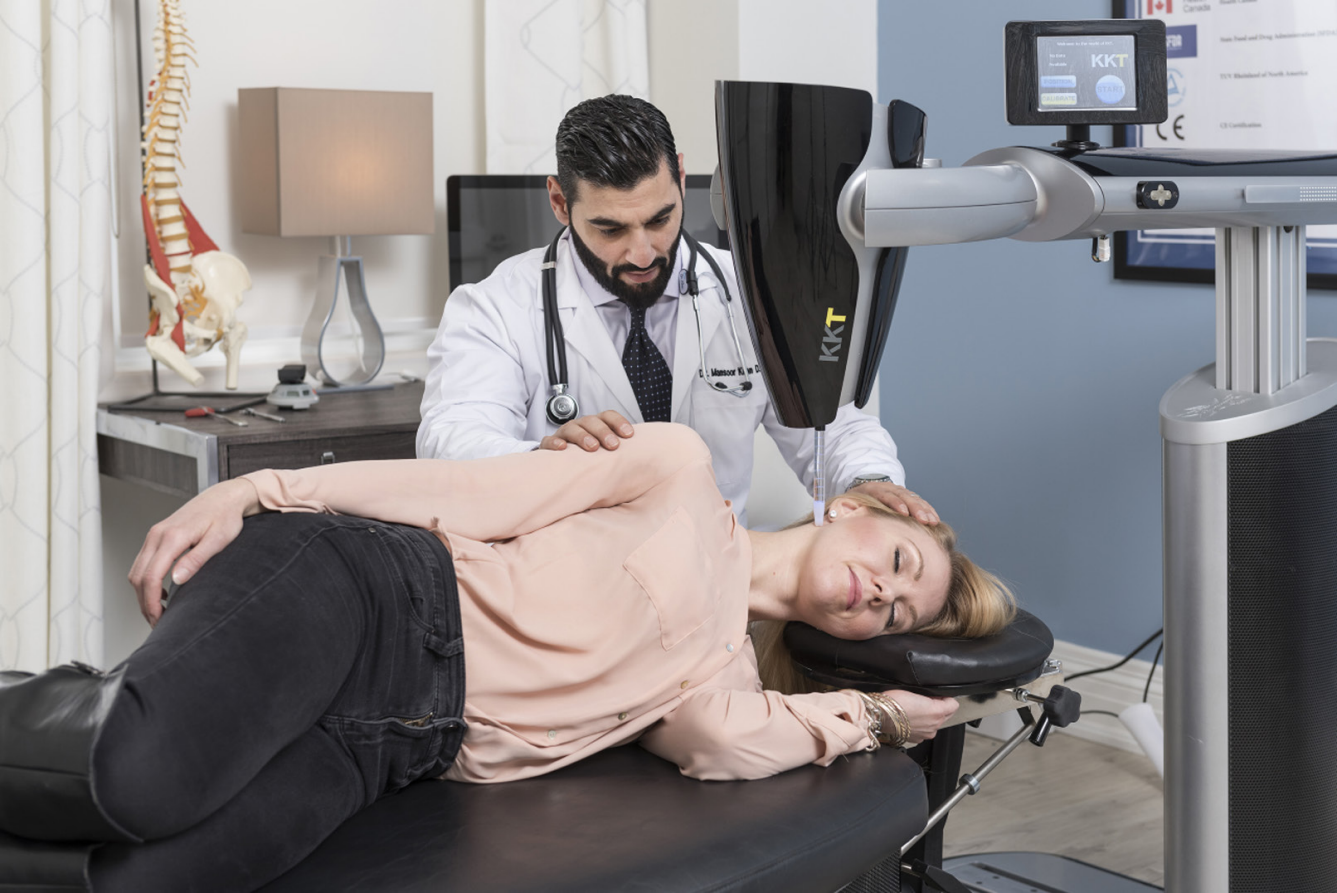
KKT device

The KKT Treatment device is a sound transducer‐driven, vibratory stylus, impulse delivery mechanism. The device head is mounted on a flexibly positionable armature on a fixed stand.^3^ The head of the device, moveable in three dimensions, is positioned by the clinician and then fixed in location at the prescribed spinal location and angle. The stylus is pressure sensitive and so provides patient safety by being collapsible if the patient moves out of position (Figure 4).^3^ The device's sound transducer generates waveforms that are transmitted through to the spine causing vibration of the vertebrae and minor stretching of the soft tissues. At the initial assessment, treatment parameters are determined from digital data captured through X‐rays of the spine. Table 1 indicates the anatomical locations at which the treatment was applied in this case and the number of pulses used during each session. Although there was mid‐thoracic pain, the treatment assumption of KKT is that application to C1 will result in realignment of the spine and thereby thoracic pain levels. Consequently, as reported in Table 1, no thoracic treatment was applied. The treatment plan for the patient in this report was for 18 sessions of KKT (3 times a week for the first 3 weeks and then about once per week). After every six sessions, the patient completed the VAS for pain. The Rolland‐Morris test was completed at the first session and after the last session. At least 6 follow‐up sessions beyond the 18 were provided with patient remaining stable.

**TABLE 1:** Location of treatment and Number of pulses^a^

Treatment location and pulses				
Tx#	Cervical	Lumbar	Sacrum/iliac	Abdomen
1	Right C1 80			
2	Right C1 80			
3	Right C1 80			
4	Right C1 80			
5	Right C1 80			
6	Right C1 80			
7	Right C1 80	L5*60	Rt PSIS 80 Lt PSIS 80	
8	Right C1 80	L5*60	Rt PSIS 80 Rt Trochanter 80	
9	Right C1 80	L5*60	Rt PSIS 80	
10	Right C1 80	L5*60	Rt PSIS 80 Lt PSIS 80	
11	Right C1 80	L5*60	Rt PSIS 80 Lt PSIS 80	LLQ 40
12	Right C1 80	L5*60	Rt PSIS 80	LLQ 40
13	Right C1 80	L5*60	Rt PSIS 80	
14	Right C1 80	L5*60	Rt PSIS 80	
15	Right C1 80 C5*40	L5*60		
16	Right C1 80	L5*60		
17	Right C1 80	L5*60		
18	Right C1 80	L5*60		

*Note:* “Right” indicates application of the stimulus from the right side, e.g., Right C1 indicates the stimulus pulses were applied on the right side of C1. L or left indicates application on the left side. Asterisk * indicates application at the midline.

Abbreviations: Lft, left; LLQ, Lateral Lower Quadrant; PSIS, Posterior superior iliac spine; Rt, right; Tx, Treatment.

^a^ Each pulse lasts about 3 s including the frequency reset.

## 5 OUTCOMES

**FIGURE 5:**
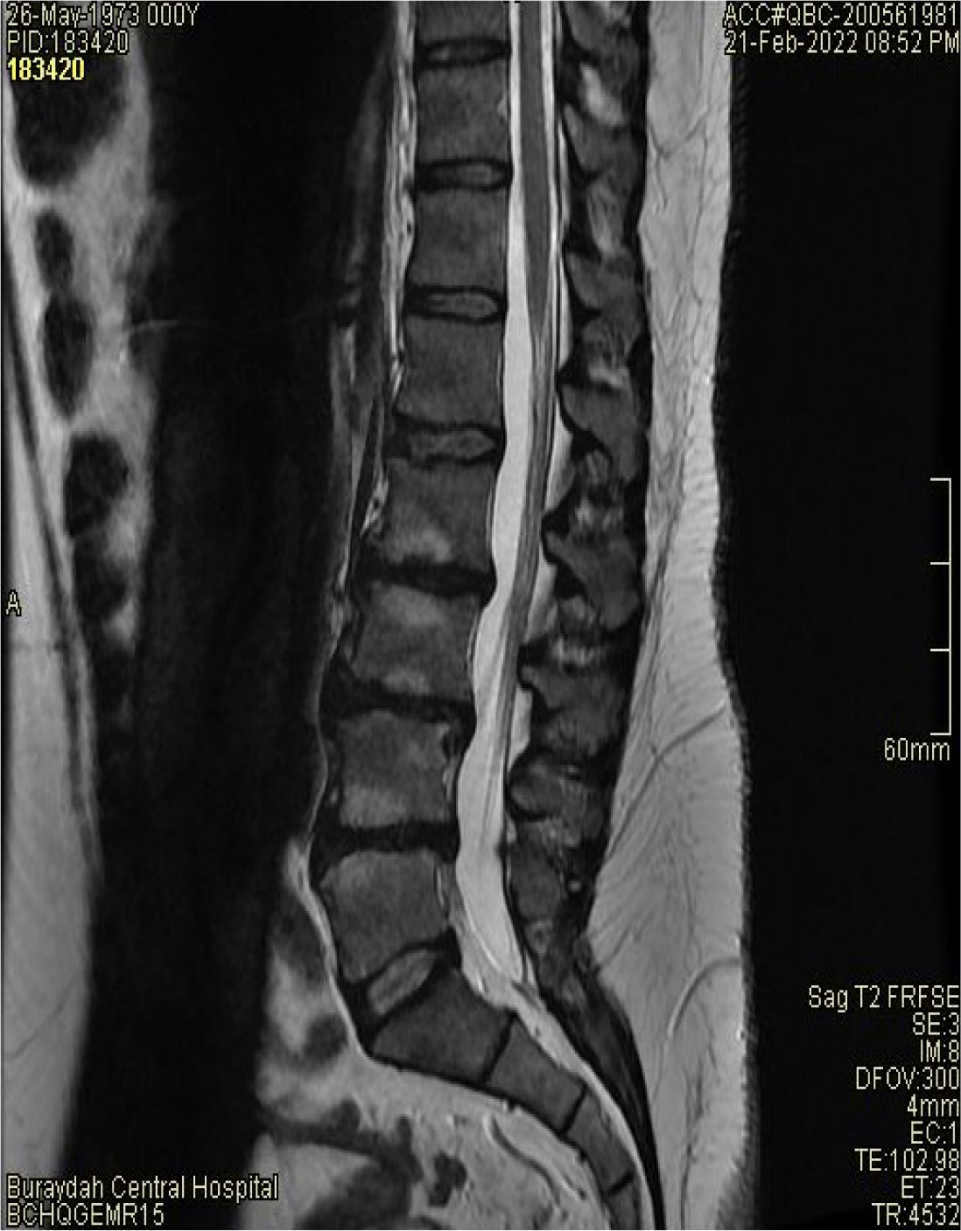
MRI of the lumbar spine after treatment showing reduced bulging of discs between L5 and L2

**FIGURE 6:**
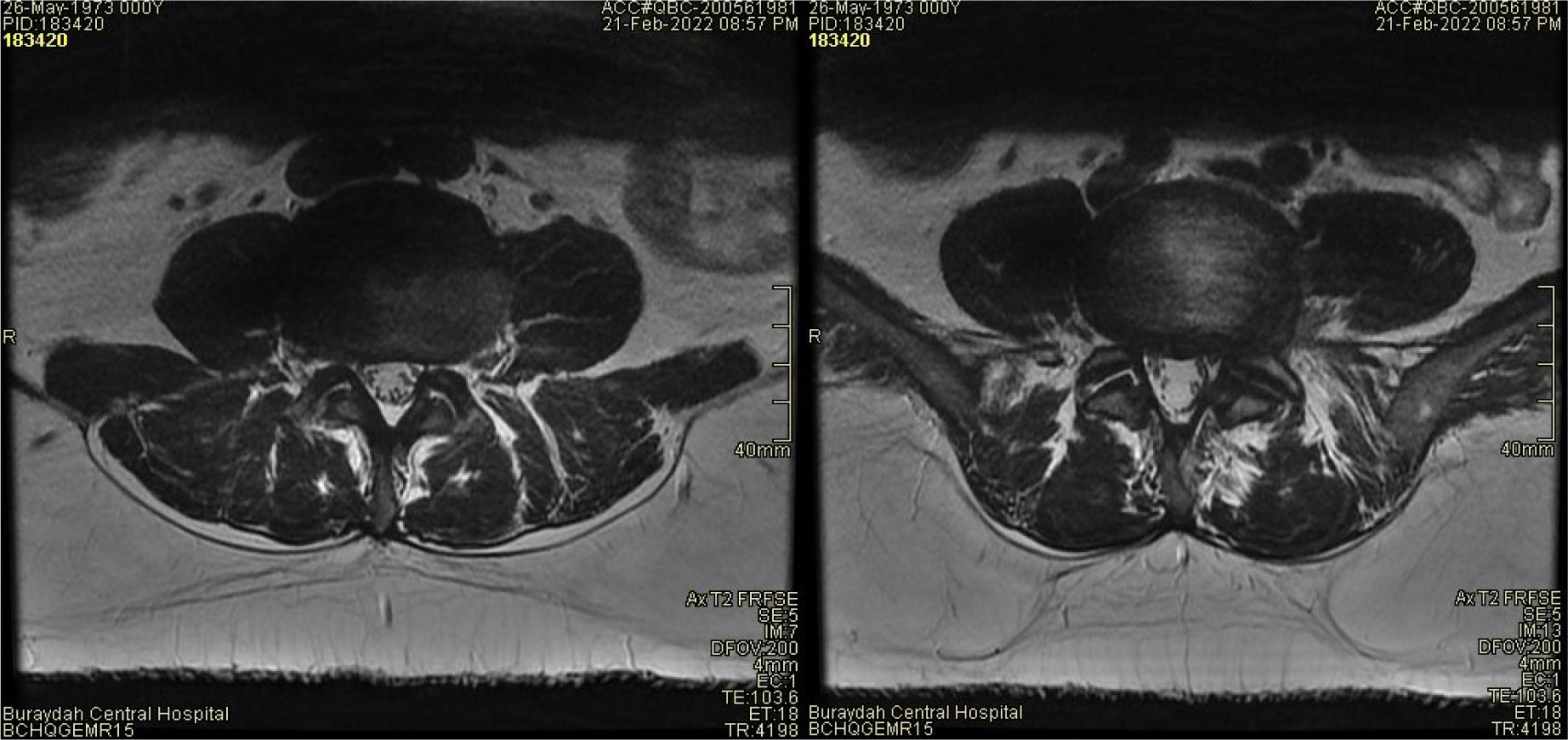
Axial MRI after treatment showing reduced bulging of discs between L5 and L3

During the course of the KKT treatments, the patient reported gradual reduction in pain. The 6 painful tender lesions at the initial assessment were reduced to 5 by session 9, to 4 by session 13, and to 0 by session 18. The VAS pain score was 9.5 at the initial assessment and was reduced to 2.5 by the final treatment session. The initial Rolland‐Morris score of 13 was reduced to 3 points by the final session. The patient reported improvement in his daily activity of life and sleep. He was also able to function at work due to his decreased pain. In addition, the patient showed a correction in clinician‐observed biomechanics of his spine and body. Shoulder and pelvic tilt steadily improved and stabilized remaining neutral from the 10th treatment onward. Cervical ROM restored to normal function at the 13th treatment as did the upper limb coordination. After the completed treatment sessions, the patient's MRI showed some resolution of L3‐L4 and L4‐L5 damage (Figures 5 and 6). No adverse events related to the treatment were experienced by the patient.

## 6 DISCUSSION

The case described in this paper demonstrates that the KKT treatment focused on LBP. Research literature reveals that even mild LBP results in significant functional loss and decreased quality of life. LBP is the second most common cause for consulting a doctor. Typical treatment of LBP includes various psychological, medical, and physical therapies. When these treatment options fail, the patient often chooses the last option—spinal surgery. However, spinal surgery may not produce symptomatic relief in the long term and so the patient returns to daily activity with complications. The KKT approach of treating the patient with sound waves,^3^, ^8^, ^9^, ^11^ presents a safe and precise alternative. This case study of a middle‐aged adult patient who presented with LBP radiating to both lower limbs treated with KKT is consistent with previously published research.^3^, ^9^, ^11^, ^12^ After the patient received 18 sessions over a period of 13 weeks, he reported improvement in disability and pain scores, sleep, mood, quality of life, and work performance, No adverse effects were observed. Improvement in the lumbar spine was noted on MRI imaging.

## 7 CONCLUSION

In conclusion, the KKT treatment for lumbar disc disorders is promising but a limitation of case reports is that they report on one or a few cases making it difficult to generalize to broad clinical practice. However, the KKT treatment results are evident in other reports and RCT results.^3^, ^9^, ^11^, ^12^, ^13^ In this case, the patient experienced symptomatic relief and, as a result, improved quality of life, mood, and work performance. Some positive structural changes were also seen on MRI. The level of improvement the patient experienced in his condition and the renewed level of confidence enabled him to return to activity without any symptoms.

## AUTHOR CONTRIBUTION

Dr. Norah Alsalamah—selection of the case, conducting the treatment, initial draft of the paper, and review of revisions. Dr. Lee Bartel—consultation on selection of case, guidance on obtaining specific case details, revision of the paper, and preparation of final version of the paper.

## FUNDING INFORMATION

The authors conducted this study in the normal course of their work related to KKT International and Neuro Spinal Innovations. There was no additional funding. Publication costs are paid by Neuro Spinal Innovations.

## CONFLICTS OF INTERESTS

The authors conducted this study in the normal course of their work related to KKT International and Neuro Spinal Innovations (NSI). They received no additional remuneration and any benefits to KKT or NSI will not affect this.

## CONSENT

Written informed consent was obtained from the patient to publish this report in accordance with the journal's patient consent policy.

## Data Availability

All basic clinical data has been reported. Physiological assessment data for all treatments and initial cervical X‐rays are available upon request from the corresponding author.
